# Longitudinal Outcomes of Bilateral and Unilateral Proximal Greater Occipital Nerve Pulsed Radiofrequency in Migraine: A Prospective Randomized Controlled Trial

**DOI:** 10.3390/medicina62081554

**Published:** 2026-08-13

**Authors:** Sukriye Dadali, Nevcihan Sahutoglu Bal, Ulku Sabuncu, Gulcin Babaoglu, Ali Costu, Hatice Babaoglan, Mustafa Cem Yilmaz, Seref Celik, Saziye Sahin, Erkan Yavuz Akcaboy

**Affiliations:** Department of Pain Medicine, Ankara Bilkent City Hospital, Ankara 06800, Türkiye

**Keywords:** migraine, greater occipital nerve, pulsed radiofrequency, neuromodulation, ultrasonography, randomized controlled trial

## Abstract

*Background and Objectives:* Greater occipital nerve pulsed radiofrequency (GON-PRF) is an effective neuromodulatory treatment for migraine; however, evidence directly comparing unilateral and bilateral approaches remains limited. This prospective randomized controlled trial compared the clinical efficacy of ultrasound-guided proximal GON-PRF performed unilaterally versus bilaterally at the C2 level in patients with migraine. *Materials and Methods*: A total of 187 patients were randomized to receive unilateral (*n* = 91) or bilateral (*n* = 96) ultrasound-guided proximal GON-PRF without concomitant local anesthetic or corticosteroids. Per-protocol analyses included 78 and 88 participants in the unilateral and bilateral groups, respectively. The primary outcome was the change in monthly headache days (MHD) from baseline to 6 months. Secondary outcomes included Visual Analog Scale (VAS) scores, acute medication days (AMD), Headache Impact Test-6 (HIT-6) scores, Migraine Disability Assessment (MIDAS) grades, and Global Perceived Effect (GPE) scores. Longitudinal outcomes were analyzed using multivariable Generalized Estimating Equations (GEE). *Results*: Of the 187 randomized participants, 166 completed the 6-month follow-up and were included in the per-protocol analysis. Both groups demonstrated significant improvements in all outcomes throughout follow-up (all *p* < 0.001). At month 6, the Bilateral Group showed lower MHD values (median [IQR]: 7.0 [6.0] vs. 10.0 [6.0], *p* = 0.030), a higher ≥50% MHD responder rate (77.3% vs. 55.1%, *p* = 0.003), and lower AMD values (*p* = 0.024). Adjusted GEE analyses demonstrated significant treatment effects favoring the Bilateral Group for average VAS (*p* = 0.047) and HIT-6 (*p* = 0.004). GPE scores also favored the Bilateral Group at month 6 (*p* = 0.044). No procedure-related complications occurred. *Conclusions*: Both unilateral and bilateral ultrasound-guided proximal GON-PRF provided significant clinical improvement in patients with migraine. However, bilateral treatment demonstrated superior longitudinal outcomes, including lower headache frequency, reduced acute medication use, higher ≥50% MHD responder rates, and greater patient-reported improvement. These findings remained consistent after adjustment for baseline covariates.

## 1. Introduction

Migraine is a highly prevalent neurological disorder and a leading cause of disability worldwide, particularly among young and middle-aged adults [1]. Contemporary epidemiological studies estimate that migraine affects approximately 14–15% of the global population, and recent Global Burden of Disease analyses indicate that its societal and economic burden continues to increase over time [2,3]. Beyond recurrent headache attacks, migraine substantially impairs daily functioning and quality of life [1]. Although pharmacological therapies remain the cornerstone of migraine management, a substantial proportion of patients exhibit insufficient therapeutic response, poor tolerability, or develop medication overuse. Consequently, minimally invasive interventional approaches targeting peripheral nociceptive pathways have attracted increasing clinical interest, particularly in patients with refractory or treatment-resistant migraine [4].

Among peripheral neuromodulatory interventions, greater occipital nerve blockade (GONB) is widely used for migraine and other primary headache disorders. Although local anesthetic-based GONB, with or without corticosteroids, may reduce headache frequency and severity, its effects are often temporary [4,5]. Pulsed radiofrequency (PRF) has emerged as a neuromodulatory alternative aimed at providing more sustained benefit. PRF delivers intermittent high-frequency electrical fields without causing destructive thermal injury and is thought to modulate nociceptive transmission through effects on both peripheral and central pain pathways [4,6]. Recent studies evaluating ultrasound-guided greater occipital nerve PRF (GON-PRF) have demonstrated favorable efficacy and safety outcomes in migraine [7,8].

Anatomical and clinical studies suggest that proximal approaches targeting the greater occipital nerve at the C2 level may provide more consistent neural targeting and sustained clinical benefit by avoiding terminal branching variability [9,10]. Randomized studies comparing distal and proximal GON-PRF techniques in migraine have demonstrated favorable outcomes with both approaches, although proximal applications may provide greater benefit in patients with more severe disease burden [10,11].

The mechanisms underlying headache relief after greater occipital nerve interventions remain incompletely understood but are thought to involve modulation within the trigeminocervical complex (TCC). Busch et al. demonstrated that unilateral occipital nerve blockade induced bilateral inhibition of nociceptive blink reflexes, supporting functional convergence between upper cervical and trigeminal nociceptive pathways [12]. Consistent with this rationale, retrospective studies evaluating unilateral and bilateral greater occipital nerve blockade reported broadly comparable outcomes in chronic migraine [13,14]. However, evidence regarding unilateral and bilateral greater occipital nerve pulsed radiofrequency (GON-PRF) applications remains limited. A recent retrospective study suggested that unilateral PRF directed to the symptomatic side may provide outcomes comparable to bilateral treatment; however, PRF was combined with repeated nerve blocks, thereby confounding the independent longitudinal effects of treatment laterality [15]. Prospective randomized data specifically evaluating unilateral versus bilateral proximal GON-PRF techniques remain scarce. Therefore, this prospective randomized controlled trial aimed to compare the clinical efficacy of unilateral and bilateral ultrasound-guided proximal GON-PRF performed at the C2 level in patients with migraine.

## 2. Materials and Methods

### 2.1. Study Design and Ethical Considerations

**Figure 1:**
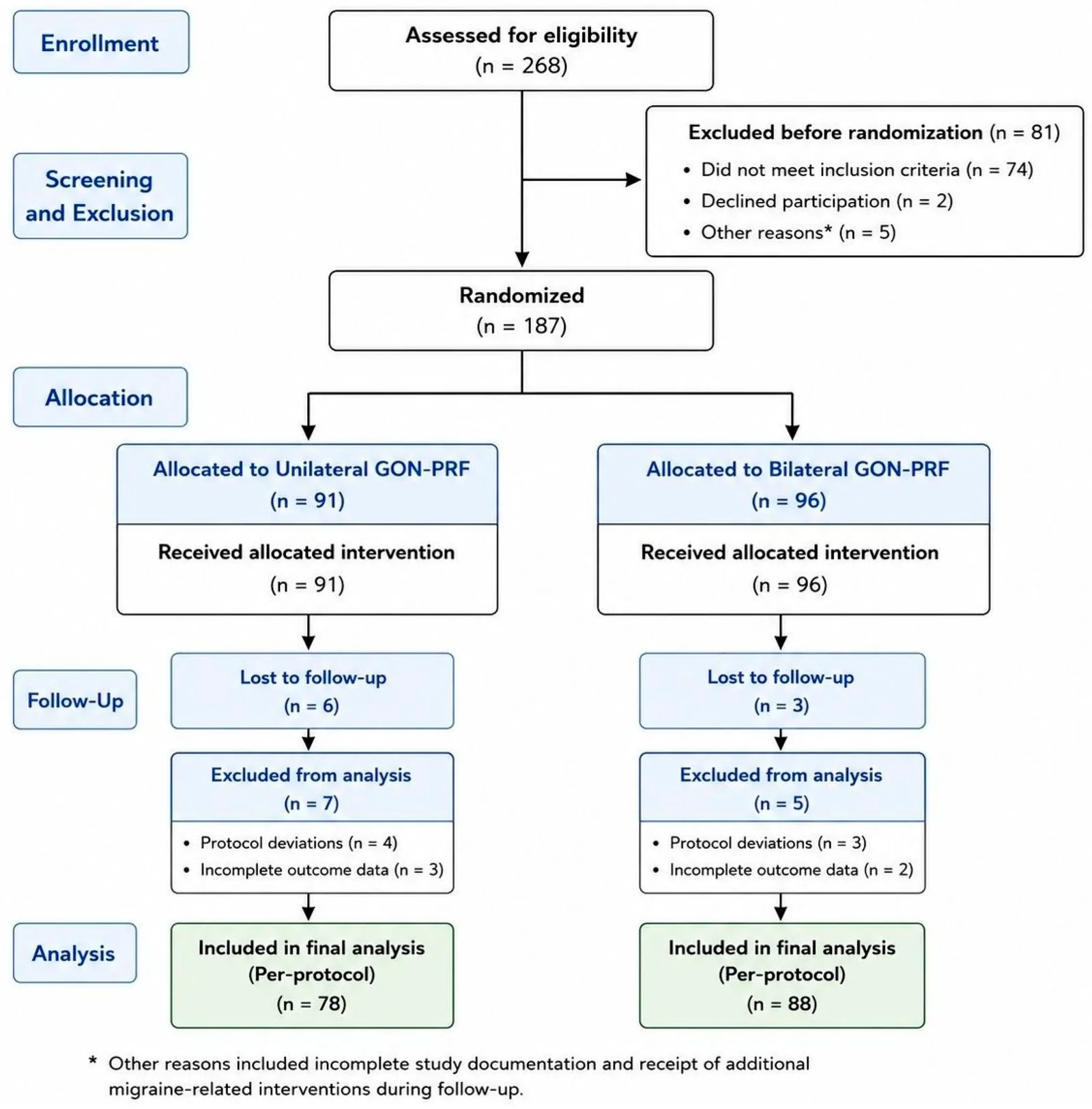
CONSORT flow diagram of participant enrollment, screening, randomization, follow-up, and per-protocol analysis. Of the 268 patients assessed for eligibility, 187 underwent randomization (91 unilateral GON-PRF: 96 bilateral GON-PRF). After follow-up and protocol-related exclusions, 166 participants (78 unilateral; 88 bilateral) were included in the final per-protocol analysis.

This single-center prospective randomized controlled trial was conducted at the Department of Pain Medicine, Ankara Bilkent City Hospital. The study protocol was approved by the Institutional Ethics Committee of Ankara Bilkent City Hospital (Decision No: TABED 1-24-763; 18 December 2024), prior to enrollment of the first participant. A subsequent protocol amendment, approved on 28 January 2026, revised the follow-up schedule from baseline, 1, 2, and 3 months to baseline, 1, 3, and 6 months to better evaluate intermediate- and longer-term clinical outcomes. No changes were made to the study design, eligibility criteria, intervention protocol, primary outcome measures, or statistical analysis plan. The study was conducted in accordance with the principles of the Declaration of Helsinki, and written informed consent was obtained from all participants prior to enrollment. The trial was registered at ClinicalTrials.gov (Identifier: NCT06894121; registration date: 18 March 2025), after participant enrollment had begun.Participant enrollment, randomization, follow-up, and analysis are summarized in the CONSORT flow diagram (Figure 1).

### 2.2. Patient Selection

Patients aged 18–65 years attending the Pain Medicine outpatient clinic were screened for eligibility. Participants fulfilling the International Classification of Headache Disorders, 3rd edition (ICHD-3) diagnostic criteria for episodic migraine (≥5 migraine attacks per month) or chronic migraine were eligible for enrollment. Patients were required to have an inadequate response to preventive migraine therapies and either be untreated with preventive medication at enrollment or have maintained a stable preventive medication regimen for at least 3 months.

Additional inclusion criteria were the absence of active infection or bleeding disorders, no current pregnancy, and no history of craniocervical surgery affecting the intervention site.

Exclusion criteria included primary headache disorders other than migraine according to ICHD-3 criteria, fewer than 5 migraine attacks per month, or secondary headache disorders such as those related to uncontrolled hypertension or intracranial pathology. Patients were also excluded if they had undergone any interventional migraine procedure, botulinum toxin injection, or non-pharmacological migraine treatment (e.g., acupuncture, physical therapy, ozone therapy, or cognitive behavioral therapy) within 3 months before the procedure. Patients who withdrew consent, missed follow-up visits, or developed exclusion criteria during the study period were excluded from the final analysis.

### 2.3. Randomization and Allocation

Participants were randomized in a 1:1 ratio to receive either unilateral or bilateral proximal GON-PRF using a computer-generated allocation sequence implemented through REDCap (Research Electronic Data Capture; Vanderbilt University, Nashville, TN, USA). The randomization sequence was generated independently before participant enrollment, and treatment allocation was performed by an independent physician using concealed allocation procedures to minimize selection bias. The treating physician was informed of the assigned intervention immediately before the procedure. Participants were aware of the intervention received because the procedure involved either unilateral or bilateral treatment. Clinical outcomes were assessed using standardized patient-reported outcome measures obtained during predefined follow-up visits, and outcome assessors were not blinded to treatment allocation. Statistical analyses were performed by an independent biostatistician who remained blinded to treatment allocation, with the study groups coded as Group 1 and Group 2 throughout the analysis.

### 2.4. Sample Size Calculation

Sample size estimation was performed using G*Power (version 3.1.9.7). Based on first-month differences in Visual Analog Scale (VAS) scores reported by Karaduman et al. [15], an a priori power analysis assuming a moderate effect size (Cohen’s *d* ≈ 0.75), a two-sided alpha level of 0.05, and 95% statistical power indicated that a minimum of 78 participants per group would be required.

### 2.5. Procedure

#### 2.5.1. GON-PRF Procedure

All procedures were performed in the operating room under sterile conditions by experienced pain specialists. Standard monitoring and intravenous access were established before the procedure. Patients were positioned prone with slight cervical flexion throughout the procedure.

**Figure 2:**
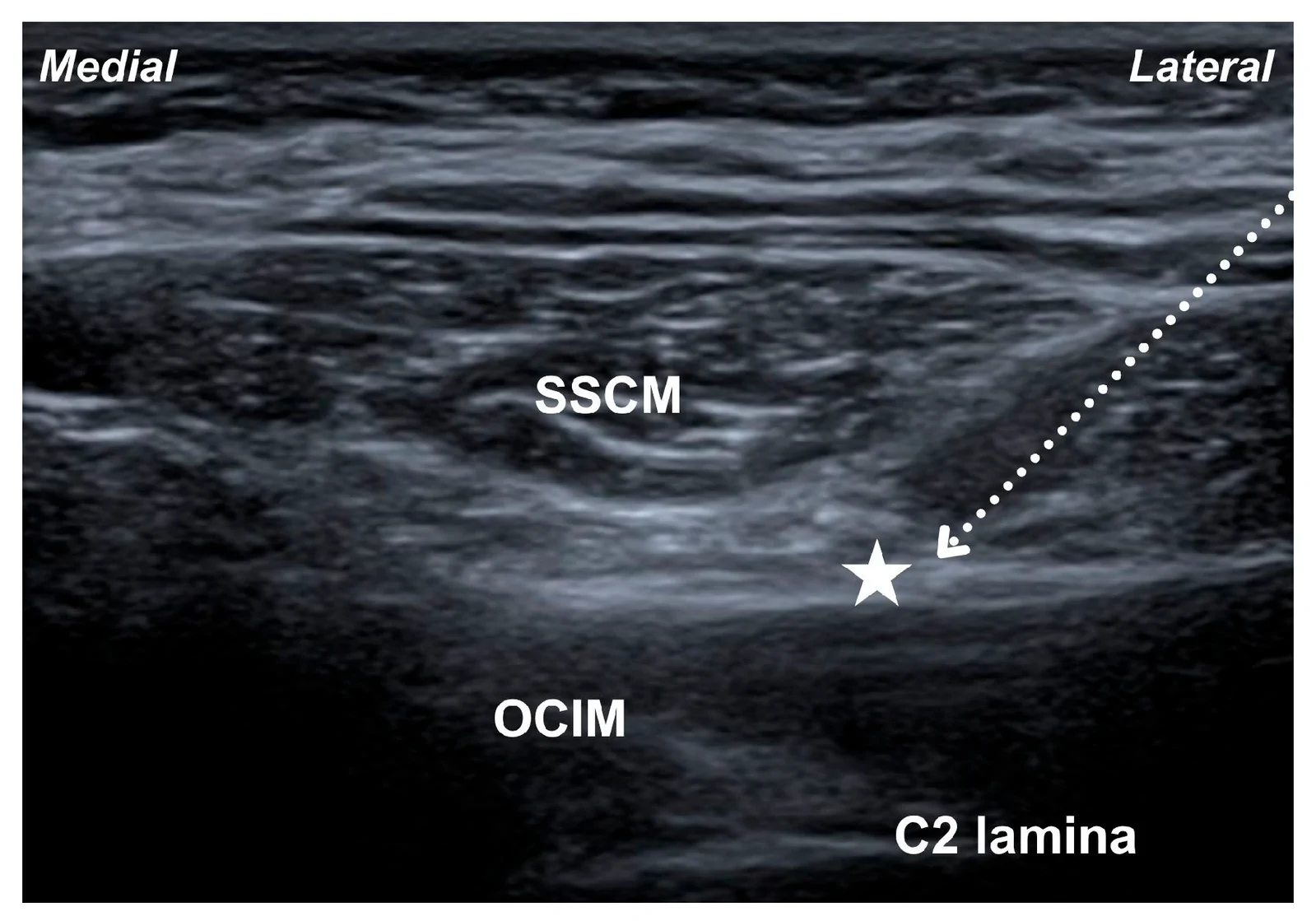
Ultrasound-guided proximal greater occipital nerve pulsed radiofrequency (GON-PRF) at the C2 level. The ultrasound image demonstrates the in-plane lateral-to-medial approach targeting the greater occipital nerve (white asterisk), located between the semispinalis capitis muscle (SSCM) and the obliquus capitis inferior muscle (OCIM), adjacent to the C2 lamina. The dotted line indicates the trajectory of the radiofrequency cannula. SSCM, semispinalis capitis muscle; OCIM, obliquus capitis inferior muscle.

Ultrasound-guided greater occipital nerve pulsed radiofrequency (GON-PRF) was performed using a proximal approach targeting the greater occipital nerve at the level of the second cervical vertebra (C2). The bifid spinous process of C2 and the obliquus capitis inferior muscle served as the principal anatomical landmarks. Following sterile skin preparation, a high-frequency linear transducer (Aplio 500; Toshiba Medical Systems, Tokyo, Japan) was placed transversely over the external occipital protuberance and advanced caudally to identify the C1–C2 vertebral levels. The transducer was subsequently moved laterally to visualize the obliquus capitis inferior and semispinalis capitis muscles. At this level, the greater occipital nerve was identified as an oval hypoechoic structure located between these muscular planes (Figure 2).

A 22-gauge, 5 cm radiofrequency cannula with a 5 mm active tip was advanced using an in-plane lateral-to-medial approach under continuous ultrasound guidance. After appropriate positioning adjacent to the target nerve, sensory stimulation testing was performed using a radiofrequency generator (NeuroTherm NT1100; NeuroTherm, Inc., Wilmington, MA, USA). Elicitation of dysesthesia or paresthesia within the greater occipital nerve dermatome at a stimulation threshold <0.3 V confirmed optimal cannula positioning.

Pulsed radiofrequency treatment was then applied for 360 s at 45 V with a pulse frequency of 5 Hz and pulse width of 5 ms while maintaining the electrode tip temperature below 42 °C. No injectate was administered during the procedure. In patients allocated to the bilateral treatment group, the same procedure was repeated contralaterally during the same session. Following the procedure, patients were monitored for at least 1 h and discharged on the same day if no complications occurred.

#### 2.5.2. Treatment Side Allocation

Patients randomized to the unilateral treatment group underwent GON-PRF on the side with predominant headache symptoms, whereas patients allocated to the bilateral treatment group received bilateral GON-PRF during the same session.

### 2.6. Outcome Measures and Data Collection

#### 2.6.1. Primary Outcome

The primary outcome measure was the change in monthly headache days (MHD) from baseline to 6 months after treatment. Monthly headache days were defined as calendar days with headache lasting at least 4 h with moderate or greater intensity, or any headache requiring acute migraine medication use. The proportion of patients achieving a ≥50% reduction in monthly headache days from baseline (MHD responder rate) at each follow-up visit was also evaluated as a predefined efficacy measure.

#### 2.6.2. Secondary Outcomes

Secondary outcome measures included headache intensity (assessed using the Visual Analog Scale [VAS]), acute medication days (AMD), headache-related impact assessed using the Headache Impact Test-6 (HIT-6), migraine-related disability assessed using the Migraine Disability Assessment (MIDAS) grade, Global Perceived Effect (GPE) score, and treatment-related adverse events.

Headache intensity was evaluated using a 10-point VAS ranging from 0 (no pain) to 10 (worst imaginable pain), reflecting average and maximum pain severity. Migraine-related disability was assessed using MIDAS categorical grades (Grade I–IV) based on total disability scores, with higher grades indicating greater disability severity (Grade I: 0–5, Grade II: 6–10, Grade III: 11–20, and Grade IV: ≥21) [16]. Headache-related functional impact was evaluated using the HIT-6 questionnaire [17]. Overall patient-reported clinical improvement was assessed using the 7-point Global Perceived Effect (GPE) scale, with higher scores indicating greater perceived benefit [18]. Scores ranged from 1 (Very much worsened, ≥75% worsening) to 7 (Very much improved, ≥75% improvement). A pain responder (MPR) was defined as a patient who achieved at least a 50% reduction in average VAS score compared with baseline.

At baseline, demographic and clinical characteristics, including migraine history, headache laterality, medication overuse, and all predefined outcome measures, were assessed before the intervention. Participants were instructed to maintain standardized headache diaries throughout the follow-up period. VAS, AMD, and GPE were assessed at baseline and 1, 3, and 6 months, whereas HIT-6 and MIDAS were evaluated at baseline, 3, and 6 months. Treatment-related adverse events were recorded throughout the follow-up period.

### 2.7. Statistical Analysis

Statistical analyses were performed using IBM SPSS Statistics version 27.0 (IBM Corp., Armonk, NY, USA). Data normality for continuous variables was evaluated using the Shapiro–Wilk test. Continuous variables were expressed as mean ± standard deviation (SD) or median (interquartile range [IQR]) according to the data distribution, while categorical variables were presented as numbers and percentages (*n*/%).

Between-group cross-sectional comparisons were performed using the independent samples *t*-test for normally distributed continuous variables and the Mann–Whitney *U* test for non-normally distributed continuous data. Categorical variables were compared using the chi-square test, Fisher’s exact test, or likelihood ratio test where appropriate. Repeated ordinal outcomes were analyzed using Friedman tests and Kendall’s coefficient of concordance. Longitudinal intragroup changes in continuous variables across time points were evaluated using Friedman tests with post hoc pairwise comparisons and Bonferroni adjustment. Effect sizes were calculated as Cramer’s *V* for categorical data, and *r* = Z/√N for Mann–Whitney *U* tests. Effect sizes were interpreted according to Cohen’s criteria (0.1: small, 0.3: moderate, and 0.5: large effect).

To evaluate longitudinal outcomes and control for potential confounding variables, multivariate Generalized Estimating Equations (GEE) modeling was applied. For count data endpoints (MHD and AMD), a GEE model utilizing a negative binomial distribution with a log-link function was constructed. For continuous endpoints (VAS and HIT-6), a linear GEE model was utilized. All GEE models included treatment group, time, and the group × time interaction term as primary independent variables, and were systematically adjusted for predefined baseline covariates: age, sex, migraine duration, baseline medication overuse, and baseline headache laterality. The primary efficacy analysis followed a per-protocol approach and included participants who completed the scheduled follow-up without major protocol deviations. Participants who received additional migraine interventions, had incomplete outcome data, or were lost to follow-up were excluded from the efficacy analysis because treatment outcomes could not be evaluated according to the predefined intervention protocol. A two-tailed *p*-value < 0.05 was considered statistically significant.

## 3. Results

### 3.1. Baseline Demographics and Clinical Characteristics

Of the 187 randomized participants, 166 completed the 6-month follow-up and were included in the final per-protocol analysis, comprising 78 patients in the unilateral treatment group and 88 patients in the bilateral treatment group.

Baseline sociodemographic characteristics, including age, sex distribution, body mass index (BMI), marital status, and educational level, were comparable between the groups (all *p* > 0.05; Table 1). Similarly, no significant between-group differences were observed regarding hypertension, diabetes mellitus, hyperlipidemia, coronary artery disease, or thyroid disorders. Asthma was more frequent in the unilateral treatment group than in the bilateral treatment group (16.7% vs. 4.5%, *p* = 0.011; Table 1).

Migraine-related baseline characteristics are summarized in Table 1. The groups did not differ significantly regarding time since migraine diagnosis, migraine subtype, duration of chronic migraine, family history of migraine, or the frequency and duration of medication overuse (all *p* > 0.05). Headache laterality distribution differed significantly between the groups (*p* < 0.001), with bilateral and switching headache patterns being more common in the bilateral treatment group, whereas unilateral headache patterns were more frequent in the unilateral treatment group.

### 3.2. Primary Outcome: Monthly Headache Days

Within-group analyses demonstrated significant reductions in MHD at all follow-up evaluations compared with baseline values in both treatment groups (all *p* < 0.001; Table 2).

Between-group comparisons showed no significant differences in absolute MHD values at baseline (*p* = 0.193), month 1 (*p* = 0.572), or month 3 (*p* = 0.163). However, at month 6, the bilateral treatment group demonstrated lower MHD values compared with the unilateral treatment group (median [IQR]: 7.0 [6.0] vs. 10.0 [6.0], *p* = 0.030, *r* = 0.17; Table 2).

**Figure 3:**
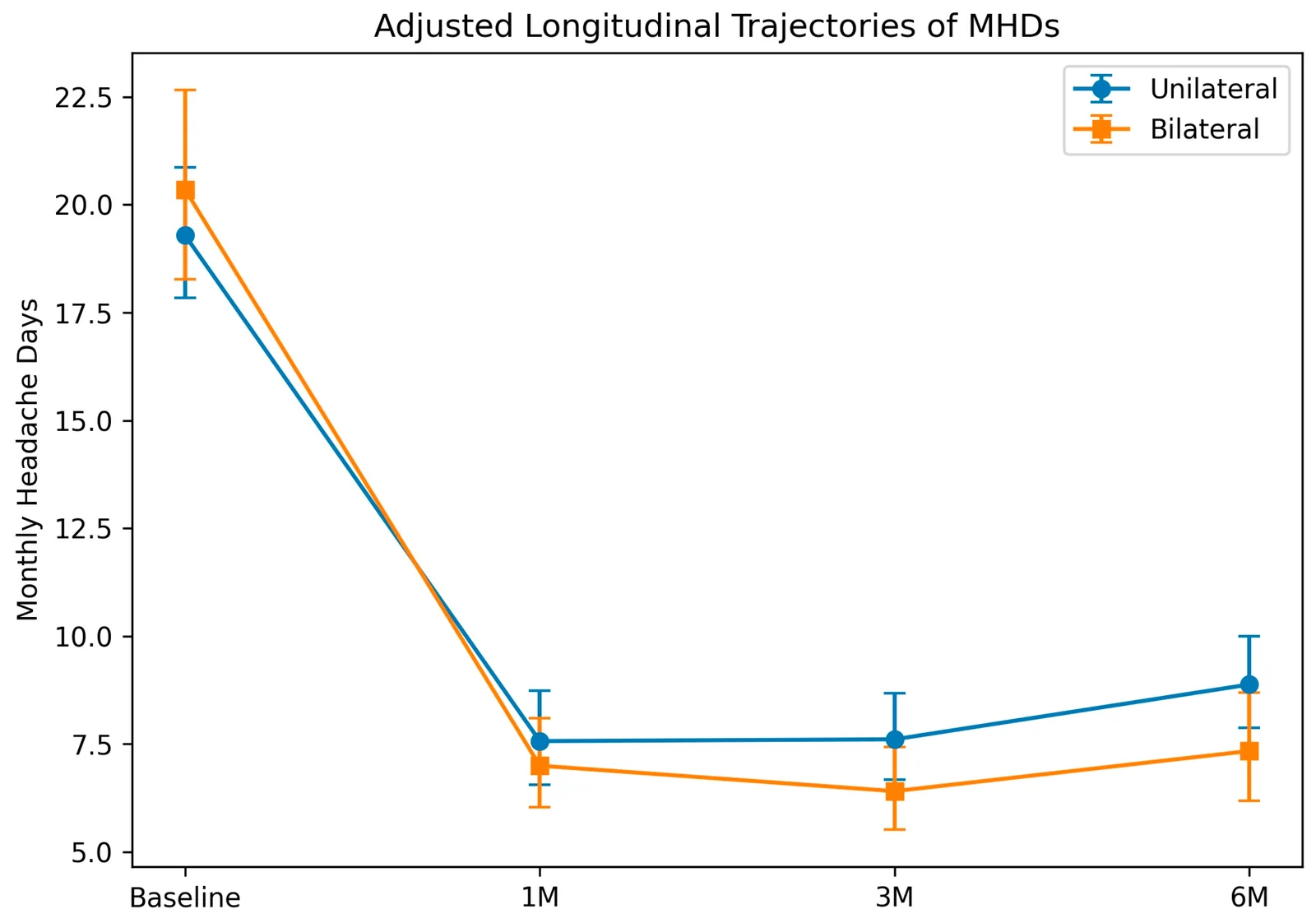
Multivariable-adjusted longitudinal trajectories of monthly headache days (MHD) over the 6-month follow-up continuum.

The reduction in MHD from baseline was comparable between groups at month 1 (*p* = 0.114), whereas greater reductions were observed in the bilateral group at months 3 and 6 (*p* = 0.030 and *p* = 0.012, respectively; Table 2). The multivariable-adjusted longitudinal trajectories of MHD are shown in Figure 3.

In the adjusted GEE model, a significant effect of time on MHD outcomes was observed (Wald χ^2^ = 561.459, *p* < 0.001). Although the group × time interaction showed borderline significance (Wald χ^2^ = 7.666, *p* = 0.053), estimated marginal mean trajectories numerically favored the bilateral treatment group throughout follow-up. Headache laterality was not independently associated with longitudinal MHD trajectories after multivariable adjustment (*p* = 0.422), whereas medication overuse remained independently associated with outcome trajectories (Wald χ^2^ = 12.663, *p* < 0.001).

The proportion of patients achieving a ≥50% reduction in MHD did not differ significantly between groups at month 1 (71.8% vs. 81.8%, *p* = 0.141) or month 3 (74.4% vs. 86.4%, *p* = 0.075). At month 6, however, responder rates were significantly higher in the bilateral treatment group compared with the unilateral treatment group (77.3% vs. 55.1%, *p* = 0.003).

### 3.3. Pain Intensity Outcomes

Baseline average and maximum VAS scores were comparable between the groups (*p* = 0.651 and *p* = 0.554, respectively; Table 2). Both treatment groups demonstrated significant reductions in average and maximum VAS scores at all follow-up evaluations compared with baseline values (all *p* < 0.001; Table 2).

Compared with the unilateral treatment group, average VAS scores were lower in the bilateral group at month 1 (*p* = 0.018, *r* = 0.18), month 3 (*p* = 0.006, *r* = 0.22), and month 6 (*p* = 0.006, *r* = 0.22; Table 2).

Maximum VAS scores did not differ significantly between groups at month 1 (*p* = 0.114), but were lower in the bilateral group at months 3 and 6 (both *p* = 0.006; Table 2).

The adjusted GEE model demonstrated a significant main effect of treatment group on longitudinal average VAS trajectories (*p* = 0.047).

The proportion of pain responders (MPR), defined as patients achieving a ≥50% reduction in average VAS score from baseline, was higher in the bilateral treatment group at month 1 (59.1% vs. 42.3%, *p* = 0.043). No significant between-group differences in pain responder rates were observed at months 3 and 6 (*p* = 0.062 and *p* = 0.100, respectively).

### 3.4. Acute Medication Days

Monthly acute medication days (AMD) decreased significantly from baseline at all follow-up evaluations in both treatment groups (all *p* < 0.001; Table 2).

AMD values were comparable between groups at baseline (*p* = 0.651) and month 1 (*p* = 0.151). However, the bilateral treatment group demonstrated lower AMD values at month 3 (*p* = 0.048, *r* = 0.13) and month 6 (*p* = 0.024, *r* = 0.17) compared with the unilateral treatment group (Table 2).

### 3.5. Headache Impact and Disability Outcomes

**Figure 4:**
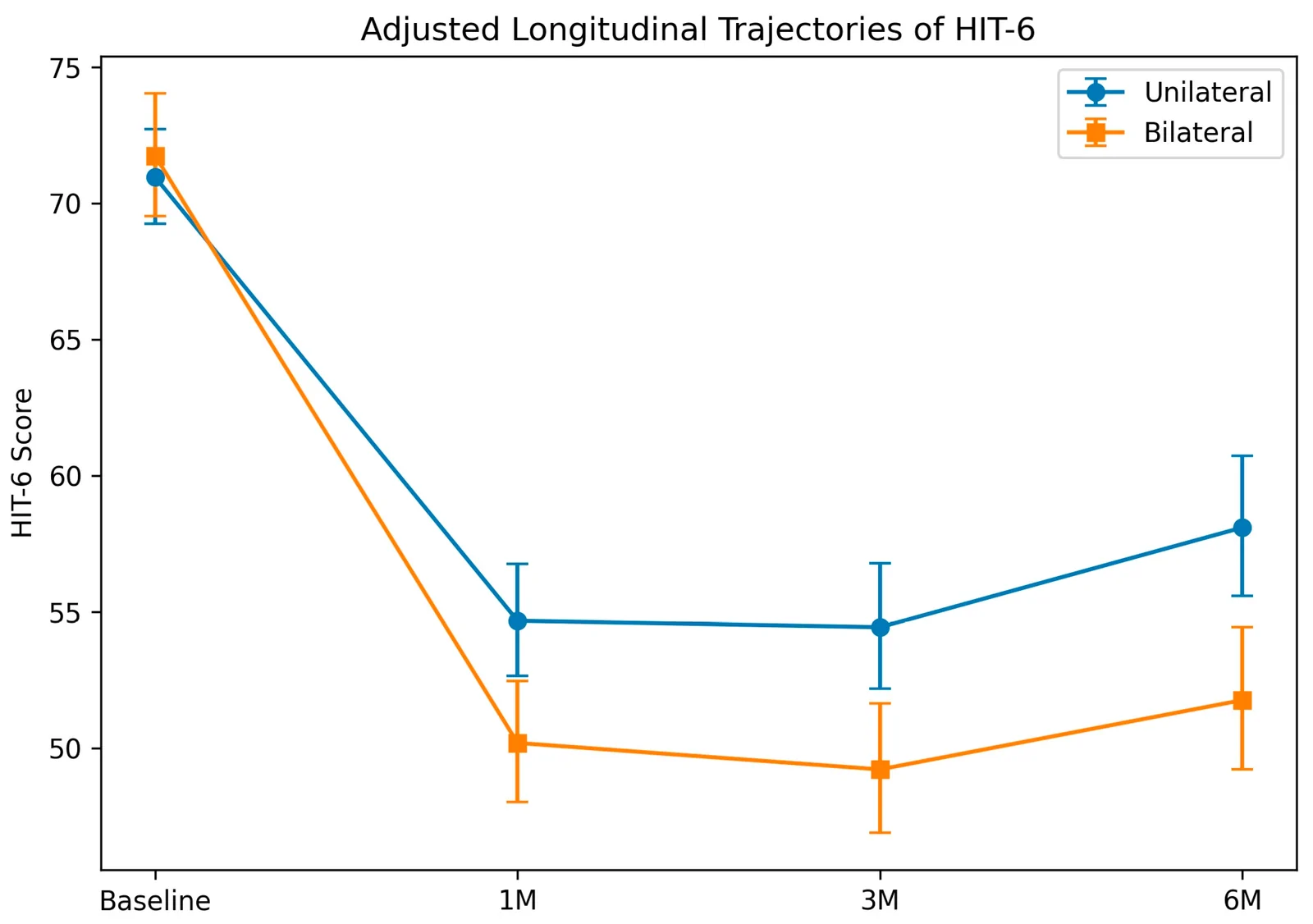
Multivariable-adjusted longitudinal trajectories of Headache Impact Test-6 (HIT-6) scores across the 6-month study timeline.

Baseline HIT-6 scores were similar between groups (*p* = 0.446; Table 3). Both treatment groups demonstrated significant reductions in HIT-6 scores throughout follow-up compared with baseline values (all *p* < 0.001). During follow-up, HIT-6 scores were consistently lower in the bilateral treatment group at months 1, 3, and 6 (all *p* < 0.001; Table 3). Adjusted longitudinal GEE analysis demonstrated significant effects of treatment group (*p* = 0.004), time (*p* < 0.001), and group × time interaction (*p* < 0.001) on HIT-6 outcomes. The multivariable-adjusted longitudinal trajectories of HIT-6 scores are presented in Figure 4.

At baseline, MIDAS disability grades differed significantly between groups (*p* < 0.001, Cramer’s *V* = 0.32), with Grade IV disability being more frequent in the bilateral treatment group (Table 3). Both groups demonstrated significant improvement in MIDAS grades during follow-up compared with baseline distributions (all *p* < 0.001). No significant between-group differences were observed in MIDAS grade distributions at month 3 (*p* = 0.114) or month 6 (*p* = 0.060; Table 3).

### 3.6. Patient-Reported Global Outcomes

Global perceived effect (GPE) score distributions showed borderline between-group differences at month 1 (*p* = 0.054) and month 3 (*p* = 0.051; Table 4). At month 6, GPE distributions differed significantly between the groups (*p* = 0.044; Table 4). A greater proportion of patients in the unilateral treatment group reported a GPE score of 4 compared with the bilateral treatment group (21.8% vs. 8.0%), whereas higher GPE score categories were more frequently observed in the bilateral treatment group (Table 4). No procedure-related complications or long-term adverse events were recorded in either group.

## 4. Discussion

To the best of our knowledge, the present study represents the first prospective randomized controlled trial directly comparing the long-term clinical efficacy of ultrasound-guided proximal greater occipital nerve pulsed radiofrequency (GON-PRF) performed via unilateral and bilateral approaches at the C2 level in patients with migraine. Both treatment groups demonstrated significant improvements across all primary and secondary outcome measures, including monthly headache days (MHD), pain intensity (VAS), headache-related impact (HIT-6), migraine-related disability (MIDAS), and acute medication days (AMD) throughout the 6-month follow-up period. Although both treatment strategies demonstrated substantial clinical benefit, several longitudinal outcome measures showed more favorable trajectories in the Bilateral Group during intermediate-to-late follow-up evaluations. At 6 months, patients treated bilaterally exhibited lower MHD values, higher ≥50% responder rates, reduced acute medication use, and better global perceived improvement compared with the Unilateral Group. These findings remained significant after adjustment for baseline medication overuse, headache laterality, and other relevant covariates.

The overall clinical improvement observed in both treatment groups is consistent with previous literature supporting the efficacy of proximal GON-PRF in migraine management [2]. Recent randomized studies comparing proximal and distal GON-PRF techniques demonstrated that both approaches are effective and generally well tolerated in patients with migraine [10,11]. Babaoğlu et al. reported that proximal GON-PRF may provide greater reductions in headache days and severe attack frequency, particularly in patients with higher baseline disease burden [10]. In contrast, Perdecioğlu et al. observed comparable clinical efficacy between proximal and distal approaches, while noting shorter procedure duration and less procedure-related discomfort with distal stimulation [11]. Taken together, these findings suggest that although both anatomical approaches appear clinically effective, proximal targeting at the C2 level may provide additional therapeutic benefit in selected clinical settings. Accordingly, the proximal technique was adopted as the standardized intervention approach in the present study.

The mechanisms underlying prolonged clinical effects of greater occipital nerve interventions are not fully understood but are thought to involve modulation within the trigeminocervical complex (TCC), where afferent inputs from the upper cervical roots and trigeminal nociceptive pathways converge [19,20,21]. Functional interactions within this network may allow unilateral greater occipital interventions to exert bilateral central effects through modulation of second-order neurons and interneuronal circuits extending from the trigeminal nucleus caudalis to the upper cervical spinal segments [12]. Supporting this concept, Busch et al. demonstrated that unilateral occipital nerve blockade induced bilateral inhibition of nociceptive blink reflex responses in healthy volunteers, providing neurophysiological evidence for functional connectivity between occipital and trigeminal nociceptive systems [12]. Systematic reviews have further emphasized that the convergence of trigeminal and upper cervical afferents within the TCC constitutes an important mechanistic basis for occipital nerve interventions in migraine [22,23]. Consistent with this neurophysiological framework, retrospective studies evaluating conventional greater occipital nerve blockade (GONB) reported broadly comparable clinical outcomes between unilateral and bilateral procedures in chronic migraine populations [13,14].

Previous studies comparing unilateral and bilateral occipital interventions in chronic migraine have largely focused on repeated GON blockade protocols rather than isolated pulsed radiofrequency neuromodulation [13,14,15]. In the retrospective studies conducted by Ünal et al. and Karaoğlan et al., repeated proximal or distal GON blocks with local anesthetics produced comparable clinical improvements after unilateral and bilateral applications during relatively short follow-up periods [13,14]. Similarly, Karaduman et al. reported no significant long-term differences between unilateral and bilateral PRF-based approaches; however, pulsed radiofrequency in that study was performed in combination with repeated conventional GON blockade sessions and additional bupivacaine administration [15]. These methodological differences may be clinically relevant, as repeated local anesthetic exposure may contribute to sustained symptom improvement and potentially obscure the independent longitudinal effects of neuromodulation itself. In contrast, the present study evaluated isolated proximal GON-PRF without concomitant local anesthetic or corticosteroid injection and demonstrated more favorable longitudinal clinical outcomes in the Bilateral Group.

An additional finding of the present study was the divergence in treatment response over time between unilateral and bilateral interventions. Although both groups demonstrated substantial early clinical improvement, several outcome measures favored the Bilateral Group at later follow-up evaluations. These observations were further supported by adjusted longitudinal GEE analyses. After adjustment for age, sex, migraine duration, baseline medication overuse, and headache laterality, significant group effects remained evident for headache intensity (VAS; *p* = 0.047) and headache-related impact (HIT-6; *p* = 0.004) over the 6-month follow-up period. Although the absolute between-group difference in median monthly headache days at 6 months was modest, bilateral treatment was associated with a substantially higher ≥50% responder rate (77.3% vs. 55.1%). Because a ≥50% responder rate is a recommended and widely used efficacy outcome in International Headache Society guidelines for preventive migraine trials, it provides an important patient-centered measure for interpreting the clinical relevance of the observed treatment differences [24].

An important consideration when interpreting these findings is the baseline imbalance in headache laterality between the treatment groups, with bilateral and side-switching headache patterns being more frequent in the Bilateral Group. Patients with bilateral or side-switching headache patterns may theoretically derive greater benefit from bilateral neuromodulation, introducing the possibility of residual confounding despite randomization. Nevertheless, headache laterality was prospectively included as a predefined covariate in all multivariable GEE analyses and was not independently associated with longitudinal MHD trajectories after adjustment. These findings suggest that the observed superiority of bilateral proximal GON-PRF cannot be explained solely by baseline headache laterality. However, residual confounding cannot be completely excluded, and future multicenter randomized trials incorporating stratified randomization according to headache laterality are warranted to further validate these findings.

One possible explanation for these findings is that bilateral neuromodulatory input may provide more extensive modulation of nociceptive processing within the trigeminocervical complex than unilateral stimulation alone. Although unilateral occipital interventions may induce bilateral central effects through functional convergence within the TCC, bilateral stimulation could potentially produce a more sustained neuromodulatory response. However, these mechanisms remain speculative and require further experimental investigation.

Several limitations should be acknowledged. First, this was a single-center study, which may limit the generalizability of the findings. Second, a sham-controlled design was not included because simulated proximal radiofrequency procedures without energy delivery were considered difficult to justify ethically and practically in our interventional setting. Third, although the sample size exceeded the minimum requirement determined during study planning, longer follow-up durations would be valuable to better define the persistence of treatment response beyond 6 months. In addition, baseline headache laterality was not fully balanced between the treatment groups despite randomization, likely reflecting a chance imbalance in this single-center trial. Although longitudinal analyses were adjusted for headache laterality and other relevant baseline covariates, and headache laterality was not independently associated with longitudinal outcomes in the multivariable GEE models, residual confounding cannot be completely excluded. Furthermore, because the study outcomes were primarily based on patient-reported measures and neither participants nor outcome assessors were blinded, the possibility of reporting bias cannot be completely excluded. Finally, because efficacy analyses followed a per-protocol approach, the potential influence of participant attrition and protocol deviations on treatment-effect estimates cannot be completely excluded. Accordingly, although the findings consistently favored bilateral proximal GON-PRF, this apparent superiority should be interpreted in light of the study limitations described above.

## 5. Conclusions

Both unilateral and bilateral ultrasound-guided proximal greater occipital nerve pulsed radiofrequency (GON-PRF) performed at the C2 level resulted in significant and clinically meaningful improvements in headache frequency, pain intensity, headache-related disability, acute medication use, and patient-reported outcomes in patients with migraine. However, bilateral proximal GON-PRF demonstrated more favorable longitudinal clinical outcomes at intermediate and late follow-up evaluations, particularly with respect to monthly headache days, ≥50% monthly headache day responder rates, acute medication consumption, and headache-related impact. Although residual confounding related to baseline headache laterality imbalance cannot be completely excluded, these findings support a potential role for bilateral proximal GON-PRF as a sustained neuromodulatory strategy in migraine management. Further multicenter studies with longer follow-up durations are warranted to confirm these findings and better define the long-term clinical role of bilateral neuromodulatory approaches in migraine treatment.

## Figures and Tables

**Table 1:** Baseline Demographics, Comorbidities, and Migraine-Specific Clinical Characteristics of the Treatment Groups.

Parameter	Unilateral Group (*n* = 78)	Bilateral Group (*n* = 88)	*p*-Value
**Sociodemographic Profiles**			
Age (years), mean ± SD	44.9 ± 9.5	44.2 ± 10.9	0.662
Gender (Female/Male), *n* (%)	61 (78.2%)/17 (21.8%)	63 (71.6%)/25 (28.4%)	0.374
Body Mass Index (BMI), median (IQR)	25.9 (5.3)	27.1 (6.8)	0.710
Marital Status, *n* (%)			0.823
Married	68 (87.2%)	75 (85.2%)	
Single	10 (12.8%)	13 (14.8%)	
**Education Level, *n* (%)**			0.753
Primary school	15 (19.2%)	24 (27.3%)	
Secondary school	7 (9.0%)	7 (8.0%)	
High school	25 (32.1%)	26 (29.5%)	
University	31 (39.7%)	31 (35.2%)	
**Comorbidities and Systemic Diseases, *n* (%)**			
Hypertension	28 (35.9%)	23 (26.1%)	0.182
Diabetes Mellitus	8 (10.3%)	11 (12.5%)	0.808
Hyperlipidemia	7 (9.0%)	9 (10.2%)	0.800
Coronary Artery Disease	1 (1.3%)	4 (4.5%)	0.372
Asthma	13 (16.7%)	4 (4.5%)	**0.011**
Thyroid Disorder	13 (16.7%)	12 (13.6%)	0.666
**Migraine-Specific Characteristics**			
Time since migraine diagnosis (years), median (IQR)	17.5 (15.3)	20.0 (20.0)	0.426
Migraine Type (Chronic/Episodic), *n* (%)	70 (89.7%)/8 (10.3%)	83 (94.3%)/5 (5.7%)	0.387
Duration of Chronic Migraine (years), median (IQR)	5.0 (8.0)	7.0 (9.0)	0.050
Family History of Migraine, *n* (%)	38 (48.7%)	49 (55.7%)	0.437
Medication Overuse Presence, *n* (%)	49 (62.8%)	65 (73.9%)	0.135
Duration of Medication Overuse (months), median (IQR)	24.0 (48.0)	36.0 (48.0)	0.512
**Location of Headache, *n* (%)**			**<0.001**
Right unilateral	16 (20.5%)	5 (5.7%)	
Left unilateral	26 (33.2%)	7 (8.0%)	
Switching side	22 (28.2%)	36 (40.9%)	
Bilateral	14 (17.9%)	40 (45.5%)	
**Active Prophylactic Medications ^a^, *n* (%)**			
None	15 (19.2%)	6 (6.8%)	—
Beta blockers	30 (38.5%)	38 (43.2%)	
SNRIs	22 (28.2%)	27 (30.7%)	
TCAs	15 (19.2%)	15 (17.0%)	
Topiramate	8 (10.3%)	14 (15.9%)	
SSRIs	11 (14.1%)	7 (7.9%)	
Flunarizine	5 (6.4%)	12 (13.6%)	
ACEIs/ARBs	3 (3.8%)	2 (2.3%)	
Valproate	2 (2.6%)	2 (2.3%)	
Lamotrigine	3 (3.8%)	0 (0.0%)	
Gabapentinoids	1 (1.3%)	0 (0.0%)	

IQR: Interquartile Range; BMI: Body Mass Index; SNRI: Serotonin-Norepinephrine Reuptake Inhibitor; TCA: Tricyclic Antidepressant; SSRI: Selective Serotonin Reuptake Inhibitor; ACEI: Angiotensin-Converting Enzyme Inhibitor; ARB: Angiotensin Receptor Blocker. Data are presented as mean ± SD or median (interquartile range [IQR]) for continuous variables, and as number/percentage (*n*/%) for categorical variables. Bold *p*-values indicate statistically significant differences (*p* < 0.05). Between-group comparisons were performed using the independent samples *t*-test for normally distributed continuous data, the Mann–Whitney *U* test for non-normally distributed continuous variables, the Chi-square test for categorical parameters, and Fisher’s exact test where cell counts were limited. ^a^ Total percentages exceed 100% because multiple prophylactic medications could be used concurrently by individual patients.

**Table 2:** Longitudinal Clinical Outcomes of the Treatment Groups Throughout Follow-Up.

Outcome Measure	Unilateral Group (*n* = 78)	Bilateral Group (*n* = 88)	Between-Group *p*-Value *	Effect Size (*r*)
**Primary Outcome**				
**Monthly Headache Days (MHD)**				
Baseline	20.0 (8.0)	20.0 (13.0)	0.193	0.10
Month 1	7.0 (5.3) ^a^	7.0 (5.8) ^a^	0.572	0.04
Month 3	7.5 (5.0) ^a^	6.5 (6.8) ^a^	0.163	0.10
Month 6	10.0 (6.0) ^a^	7.0 (6.0) ^a,b^	**0.030**	0.17
**Secondary Outcomes**				
**Average VAS Score**				
Baseline	8.0 (1.0)	8.0 (2.0)	0.617	—
Month 1	5.0 (2.0) ^a^	4.0 (2.0) ^a,b^	**0.018**	0.18
Month 3	4.5 (3.0) ^a^	4.0 (2.0) ^a,b^	**0.006**	0.22
Month 6	5.0 (3.0) ^a^	4.0 (3.0) ^a,b^	**0.006**	0.22
**Maximum VAS Score**				
Baseline	10.0 (0.0)	10.0 (2.0)	0.554	—
Month 1	6.0 (2.0) ^a^	6.0 (3.0) ^a^	0.114	—
Month 3	6.1 (3.0) ^a^	6.0 (3.0) ^a,b^	**0.006**	0.18
Month 6	7.0 (2.0) ^a^	6.0 (3.8) ^a,b^	**0.006**	0.23
**Monthly Acute Medication Days (AMD)**				
Baseline	15.0 (12.0)	15.0 (10.0)	0.651	0.04
Month 1	5.0 (5.0) ^a^	4.5 (4.0) ^a^	0.151	0.10
Month 3	5.0 (5.0) ^a^	4.0 (4.0) ^a,b^	**0.048**	0.13
Month 6	6.0 (6.0) ^a^	5.0 (5.0) ^a,b^	**0.024**	0.17

AMD: Monthly acute migraine medication days, MHD: Monthly Headache Days. Data are expressed as median (interquartile range [IQR]). Bold *p*-values indicate statistical significance (*p* < 0.05). * Computed via Mann–Whitney *U* test for cross-sectional between-group comparisons. ^a^ *p* < 0.001 indicates statistically significant longitudinal intragroup improvement compared to baseline values, evaluated via Friedman analysis with post hoc pairwise comparisons and Bonferroni adjustment. ^b^ *p* < 0.05 indicates statistically significant superiority of the Bilateral Group compared with the Unilateral Group at that specific cross-sectional post-procedure follow-up timepoint.

**Table 3:** Comparison of Migraine Disability (MIDAS) and Headache Impact (HIT-6) Outcomes Between Treatment Groups.

Outcome Measure	Unilateral Group (*n* = 78)	Bilateral Group (*n* = 88)	Between-Group *p*-Value	Effect Size
MIDAS Grade				Cramer’s * V *
**Baseline**			**<0.001** *	0.32
Grade II	—	2 (2.3%)		
Grade III	27 (34.6%)	14 (15.9%)		
Grade IV	51 (65.4%)	72 (81.8%)		
**Month 3**			0.114 *	0.18
Grade I	18 (23.1%) ^a^	21 (23.9%) ^a^		
Grade II	27 (34.6%) ^a^	44 (50.0%) ^a^		
Grade III	31 (39.7%) ^a^	21 (23.9%) ^a^		
Grade IV	2 (2.6%) ^a^	2 (2.3%) ^a^		
**Month 6**			0.060 *	0.21
Grade I	11 (14.1%) ^a^	20 (22.7%) ^a^		
Grade II	26 (33.3%) ^a^	38 (43.2%) ^a^		
Grade III	31 (39.7%) ^a^	26 (29.5%) ^a^		
Grade IV	10 (12.8%) ^a^	4 (4.5%) ^a^		
**HIT-6 Score**				***r* (Effect Size)**
Baseline	76.0 (12.0)	77.0 (12.0)	0.446 †	0.05
Month 1	60.0 (12.0) ^a^	48.0 (12.0) ^a,b^	**<0.001** †	0.24
Month 3	60.0 (16.5) ^a^	48.0 (19.5) ^a,b^	**<0.001** †	0.25
Month 6	60.0 (18.0) ^a^	51.0 (19.5) ^a,b^	**<0.001** †	0.25

MIDAS: Migraine Disability Assessment; HIT-6: Headache Impact Test-6. Data are expressed as number/percentage (*n*/%) for categorical MIDAS grades and median (interquartile range [IQR]) for continuous HIT-6 scores. Bold *p*-values indicate statistical significance (*p* < 0.05). * Computed via Fisher’s exact test or Chi-square test for categorical grade distributions between groups. † Computed via Mann–Whitney *U* test for cross-sectional continuous score comparisons between groups. ^a^ *p* < 0.001 indicates statistically significant longitudinal intragroup improvement over time compared to baseline values, evaluated using Related-Samples Kendall’s Coefficient of Concordance (for MIDAS) or Friedman analysis (for HIT-6) with post hoc pairwise comparisons and Bonferroni adjustment. ^b^ *p* < 0.05 indicates statistically significant superiority of the Bilateral Group compared with the Unilateral Group at that specific post-procedure cross-sectional follow-up timepoint.

**Table 4:** Distribution of Global Perceived Effect (GPE) Scores Between Treatment Groups Throughout Follow-Up.

Follow-Up Timeline and GPE Score	Unilateral Group (*n* = 78)	Bilateral Group (*n* = 88)	Between-Group *p*-Value *	Effect Size (Cramer’s *V*)
**GPE at Month 1**			0.054	0.20
Score 4 (No change)	7 (9.0%)	1 (1.1%)		
Score 5 (Slightly improved)	11 (14.1%)	8 (9.1%)		
Score 6 (Much improved)	25 (32.1%)	35 (39.8%)		
Score 7 (Very much improved)	35 (44.9%)	44 (50.0%)		
**GPE at Month 3**			0.051	0.21
Score 4 (No change)	8 (10.3%)	2 (2.3%)		
Score 5 (Slightly improved)	17 (21.8%)	14 (15.9%)		
Score 6 (Much improved)	32 (41.0%)	36 (40.9%)		
Score 7 (Very much improved)	21 (26.9%)	36 (40.9%)		
**GPE at Month 6**			**0.044**	0.22
Score 4 (No change)	17 (21.8%)	7 (8.0%) ^b^		
Score 5 (Slightly improved)	24 (30.8%)	26 (29.5%)		
Score 6 (Much improved)	21 (26.9%)	26 (29.5%)		
Score 7 (Very much improved)	16 (20.5%)	29 (33.0%) ^b^		

Data are expressed as number/percentage (*n*/%). Bold *p*-value indicates statistical significance (*p* < 0.05). GPE: Global Perceived Effect. * Computed via Likelihood Ratio test for categorical distribution comparisons between groups. ^b^ *p* < 0.05 indicates a statistically significant difference in category distribution, highlighting that fewer patients in the Bilateral Group reported “no change” (Score 4) and a higher proportion maintained maximum clinical satisfaction (Score 7) at the 6-month evaluation.

## Data Availability

The datasets generated and analyzed during the current study are not publicly available due to ethical and privacy restrictions but are available from the corresponding author on reasonable request.

## Abbreviations

The following abbreviations are used in this manuscript:

AMD	Acute Medication Days
GEE	Generalized Estimating Equations
GON	Greater Occipital Nerve
GON-PRF	Greater Occipital Nerve Pulsed Radiofrequency
GPE	Global Perceived Effect
HIT-6	Headache Impact Test-6
ICHD-3	International Classification of Headache Disorders, 3rd Edition
IQR	Interquartile Range
MHD	Monthly Headache Days
MIDAS	Migraine Disability Assessment
MPR	Meaningful Pain Relief
MOH	Medication Overuse Headache
REDCap	Research Electronic Data Capture
SD	Standard Deviation
TCC	Trigeminocervical Complex
VAS	Visual Analog Scale
